# Prevalence of Chronic Kidney Disease Among Elderly Patients in the Northeastern Region of Romania: A Descriptive, Cross-Sectional, Retrospective Analysis

**DOI:** 10.7759/cureus.80412

**Published:** 2025-03-11

**Authors:** Ionut Nistor, Ana-Maria Turcu, Anca Elena Stefan, Alexandra Covic, Bogdan Agavriloaei, Gheorghe Mahu, Ioana Alexa, Adrian Covic

**Affiliations:** 1 Department of Medicine, University of Medicine and Pharmacy Grigore T. Popa, Iași, ROU; 2 Department of Nephrology, Dr. C.I. Parhon Clinical Hospital, Iași, ROU; 3 Department of Geriatrics, Dr. C.I. Parhon Clinical Hospital, Iași, ROU; 4 Department of Cardiology, Institute of Cardiovascular Diseases, Prof. Dr. George I.M. Georgescu, Iași, ROU; 5 Department of Statistics, University of Medicine and Pharmacy Grigore T. Popa, Technology Transfer Center MAVIS, Iași, ROU

**Keywords:** ckd and elderly, ckd and romania and elderly, ckd romania, prevalence ckd elderly, prevalence ckd romania

## Abstract

In Romania, data pertaining to the incidence and prevalence of chronic kidney disease (CKD) are scant, with an even more limited understanding of the elderly demographic. In this study, we used a descriptive cross-sectional retrospective approach, drawing data from the Geriatrics and Gerontology Clinic at Dr. C.I. Parhon Clinical Hospital in Iași, Romania. We assessed renal function through creatinine measurements and estimated glomerular filtration rate (eGFR) computed using the CKD-Epidemiology Collaboration (EPI) 2021 equation. The final analysis incorporates 1080 patients, categorized based on their eGFR. The average age was 79.76 years. CKD stages 3a-5 prevalence was 25.03% (n=780) and CKD stages 1-5 was 34.6% (n=1080). The most prevalent stage was stage 3a with a frequency of 45% (n=486). Regarding age distribution, 26.2% (n=283) were young elderly, 52.1% (n=563) were elderly, and 21.6% (n=234) were old elderly, with CKD stage 3a prevalent in all age groups. This is the first study in Romania conducted exclusively on elderly patients with CKD. This study was conducted in a university center serving a population of 4.5 million patients, featuring a dedicated nephrology clinic. This study delves into the prevalence of CKD within the elderly demographic, examining its correlation with essential biomarkers of malnutrition. This study spans a decade, from 2012 to 2022, focusing on Romania's northeastern region, specifically Moldova. This is the second substantial analysis carried out in Romania and Eastern Europe, with a unique emphasis on patients aged 65 years and older.

## Introduction

Chronic kidney disease (CKD) is a global health issue affecting more than 10% of the general population, impacting over 800 million individuals worldwide. This presents a growing public health challenge, as CKD is associated with significant morbidity, mortality, and substantial healthcare costs [1]. Cardiovascular disease is the leading cause of death among CKD patients, with 40-50% of all CKD-related deaths attributed to cardiovascular causes, compared to 26% in individuals with normal kidney function [2]. Additionally, the rate of hospitalization among CKD patients is 60% higher when cardiovascular disease is present compared to those without cardiovascular complications [2].

A systematic review and meta-analysis published in 2016 provided data on the global prevalence of CKD, estimating that in Europe, the prevalence of CKD is approximately 18.4% for stages 1-5 and 11.9% for stages 3-5. The study also indicates that CKD is more prevalent in women compared to men, and the prevalence increases with age [3]. When discussing the prevalence of CKD among European countries, available data shows that CKD (stages 1-5) varies from 3.3% in Norway to 17.3% in Northeast Germany. The prevalence of mild-to-moderate CKD (stages 3-5) varies from 1% in Central Italy and 5.9% in Northeast Germany, irrespective of associated comorbidities, such as diabetes, hypertension, and obesity [4].

Age is a significant contributor to the cumulative risk associated with CKD. Elderly patients with CKD experience a greater impact from its consequences, raising concerns about poor prognosis and increased medical expenses [5-7]. According to EUROSTAT reports from 2019, there has been a decline in birth rates and an increase in life expectancy since 2010, resulting in a demographic shift towards an older population. This change has led to a notable increase in CKD prevalence, with global rates rising from 9.1% in 2017 to a significant 14.3% in 2023 [5,8,9].

In Romania, data on the incidence and prevalence of CKD are limited, with no available information concerning the elderly population. A study published in 2012 reported a CKD prevalence of 7.3% for stages 1-5, with a higher prevalence in women (9.32%) compared to men (4.85%); this estimated prevalence rate in Romania appears lower than the European average [10].

Given the scarcity of data on CKD prevalence in Romania, particularly among the elderly, the objective of this study is to determine the prevalence of chronic kidney disease in a specialized geriatric hospital population (aged >65 years) from the northeastern region of Romania (Moldova) - one of the most socioeconomically deprived regions in Europe, over the past decade [11].

## Materials and methods

Study design

We conducted a descriptive, cross-sectional, retrospective analysis. Data for this analysis were extracted from the database of the Geriatrics and Gerontology Clinic at Dr. C.I. Parhon Clinical Hospital in Iași, located in northeastern Romania, after obtaining the necessary consent. We determined the mean age of the participants and measured levels of hemoglobin, total cholesterol, total proteins, and kidney function using serum creatinine levels. The estimated glomerular filtration rate (eGFR) was calculated using the CKD-Epidemiology Collaboration (EPI) 2021 equation based on creatinine levels. CKD stages were correlated with gender and other comorbidities, and the trend of CKD was observed over the study period. For analyzing the data, we used SPSS version 19.0.1 for Windows (Armonk, NY: IBM Corp.), R (version 3.1.2) package (Vienna, Austria: R Core Team) for statistical analysis, and STATA 13 (College Station, TX: StataCorp LLC). The primary outcomes were as follows: overall prevalence of CKD, stage-based prevalence of CKD, trend of CKD over a 10-year period, and CKD prevalence based on age and gender. Secondary outcomes included hemoglobin, cholesterol, and protein levels. As this is a retrospective cross-sectional study without therapeutic intervention, approval from the ethics committee was not required.

Population

The cohort included 5124 patients aged 65 years or older. These patients were hospitalized at the Geriatrics and Gerontology Clinic of Dr. C.I. Parhon Clinical Hospital in Iași, Romania, between December 2011 and June 2022. This clinic is the only dedicated service for geriatric populations in the region, excluding palliative and psychiatric care. It is important to note that during this period, some patients required multiple evaluations. We included patients above 65 years who were evaluated in the geriatrics and gerontology clinic over a period of 10 years. We collected all laboratory measurements of plasma creatinine and urine albumin-to-creatinine (UACR) ratio. We excluded tests conducted during emergency room visits to avoid results influenced by acute illness.

Chronic kidney disease (CKD) was classified following the Kidney Disease: Improving Global Outcomes (KDIGO) criteria. Patients were categorized as having CKD if they had a prior creatinine measurement indicating an estimated glomerular filtration rate (eGFR) below 60 mL/min/1.73 m^2^ within the three months preceding their inclusion in the study. For CKD stages 1 and 2, patients were included if proteinuria was detected in their urine sediment evaluation. Additionally, it is worth mentioning that this clinic serves as the primary geriatric facility in the Moldova region. The hospital service area includes a population of approximately 4.5 million people. Admissions to the Geriatric and Gerontology Clinic are electively planned. The hospital also hosts a specialized tertiary nephrology clinic, serving the broader Moldova region. A recent report from our institution indicated that 60% of admissions were from Iași, the largest city in the county, while 40% originated from other areas within the Moldova region.

Laboratory data

The patients included in the study were categorized according to chronological age as follows: 65-74 years (young elderly), 75-84 years (adult elderly), and over 85 years (old elderly). The laboratory at Dr. C.I. Parhon Clinical Hospital is standardized according to RENAR ISO 15189:2023.

Assessment of kidney function

The normal range of creatinine was between 0.60 and 1.20 mg/dL. Given the high disease burden in elderly patients, we identified those with an eGFR <60 mL/min/1.73 m^2^ based on two determinations taken at least three months apart. For participants with an eGFR >60 mL/min/1.73 m^2^, we classified those with proteinuria on urinalysis as CKD patients.

Assessment of hemoglobin, cholesterol, and protein levels

Laboratory evaluation included assessing hemoglobin (normal values: 11.5-15.7 g/dL), total cholesterol (normal values: 120-200 mg/dL), and total protein levels (normal values: 66-87 g/L). These measurements were taken from venous blood during the initial evaluation and were interpreted based on our laboratory's standard reference intervals.

## Results

**Figure 1:**
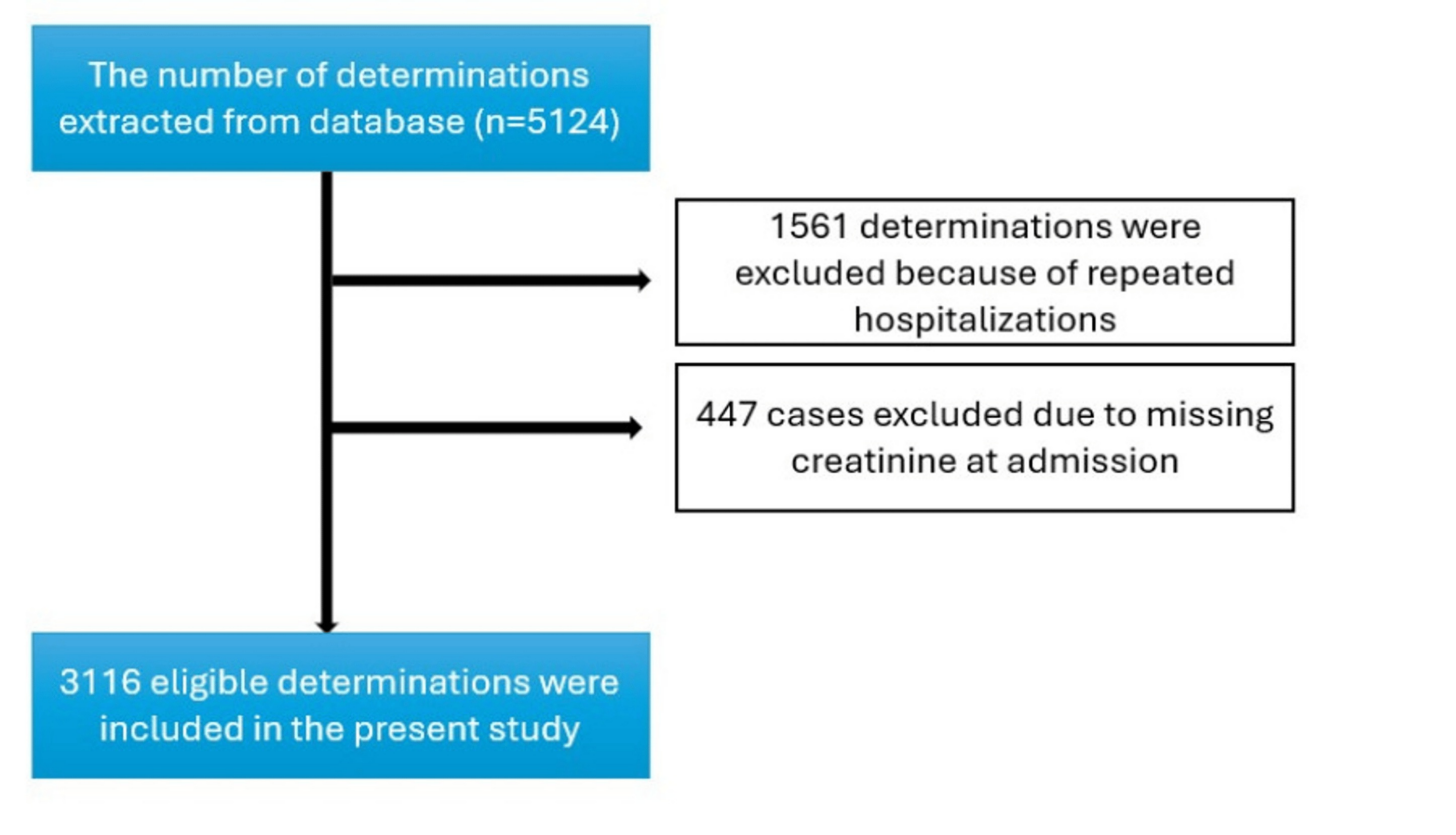
Flowchart of the study.

Out of the total 5124 determinations, 1561 were excluded due to patients requiring repeated hospitalizations. Additionally, 447 patients did not have creatinine levels measured at admission. The final analysis included 3116 patients, who were categorized based on their estimated glomerular filtration rate (eGFR) using CKD-EPI 2021 equation (Figure 1). A total of 780 patients had an eGFR ≤60 mL/min/1.73 m^2^. Given that our cohort included elderly patients with multiple comorbidities, we considered an eGFR ≤60 mL/min/1.73 m^2^ as CKD. For participants with an eGFR >60 mL/min/1.73 m^2^, we classified those with proteinuria as having CKD stages 1 or 2, totaling 300 patients. Therefore, the final analysis included 1080 patients. The mean age of the patients was 79.8 years.

Primary outcomes

Analysis of Chronic Kidney Disease Status

After analyzing the data, it was found that 25.03% (n=780) out of the 3116 patients had an estimated glomerular filtration rate (eGFR) below 60 mL/min/1.73 m^2^, while 74.9% (n=2336 patients) had an eGFR above 60 mL/min/1.73 m^2^, i.e., a prevalence of 25.03% for CKD stages 3-5 among patients above 65 years. When including CKD stages 1 and 2 (defined by the presence of proteinuria), the prevalence of CKD (stages 1-5) in this population is 9.6% (n=3116). The total number of CKD patients is 1080, out of which 780 are CKD stages 3-5 (72.2% out of the total number of CKD patients, n=1080), and 300 patients with CKD stages 1 and 2 (27.8% out of the total number of CKD patients, n=1080).

Trend of Chronic Kidney Disease (CKD) Over the 10-Year Period

**Figure 2:**
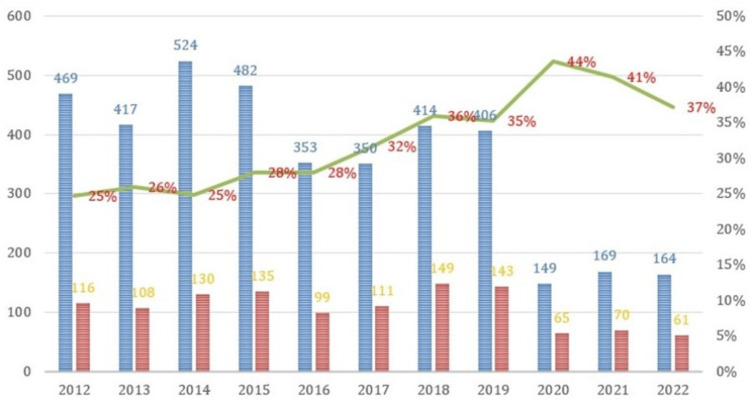
Trend of CKD over a 10-year period. Blue: eGFR >60 mL/min/1.73 m^2^; red: eGFR ≤60 mL/min/1.73 m^2^; green: eGFR ≤60 mL/min/1.73 m^2^ CKD: chronic kidney disease; eGFR: estimated glomerular filtration rate

Cases with eGFR <60 mL/min/1.73 m^2^ exhibited fluctuating patterns throughout the analyzed 10-year period, with relatively stable values initially, followed by a peak in 2018-2019. However, from 2020 onwards, when the COVID-19 pandemic became prominent in Romania, the ward was utilized as a support sector for treating COVID-19 patients. As a result, data from this period are insufficient, given the significant decrease in the number of hospitalized patients (Figure 2).

Chronic Kidney Disease and Age Distribution

When considering CKD stage and age distribution in young elderly, adult elderly, and old elderly individuals, CKD stage 3a individuals was the most prevalent when considering eGFR <60 mL/min/1.73 m^2 ^(n=283 in young elderly {26.2%}, n=563 in adult elderly {52.1%}, n=234 in old elderly {21.6%}, respectively) (Table 1).

**Table 1:** CKD stage and age distribution. CKD: chronic kidney disease

CKD stages	Young elderly (65-74 years)		Adult elderly (75-84 years)		Old elderly (over 85 years)		Total	
	N	%	N	%	N	%	N	%
1	52	18.3%	28	4.97%	0	0%	80	7.41%
2	51	18%	125	22.2%	44	18.8%	220	20.37%
3a	112	39.5%	265	47%	109	46.5%	486	45%
3b	54	19%	111	19.7%	69	29.4%	234	21.6%
4	11	3.8%	33	5.8%	10	4.2%	54	5%
5	3	1%	1	0.1%	2	0.8%	6	0.56%
Total	283	26.2%	563	52.1%	234	21.6%	1080	-

Chronic Kidney Disease and Gender

In the analyzed cohort, it was observed that during the analyzed period, there were more female patients admitted. Based on the gender distribution and estimated glomerular filtration rate (eGFR), there were more females with CKD than males (63.9% {n=690} versus 36.1% {n=390}, respectively). Regarding stages of CKD, stage 3a was the most prevalent in both genders (43% in females {n=297} and 48.4% in males {n=189}) (Table 2).

**Table 2:** Gender-based prevalence and impact in CKD. CKD: chronic kidney disease

CKD stage	Gender	
	Female (N=690) 63.9%	Male (N=390) 36.1%
Stage 1	47 (6.8%)	33 (8.4%)
Stage 2	142 (20.5%)	78 (20%)
Stage 3a	297 (43%)	189 (48.4%)
Stage 3b	163 (23.6%)	71 (18.2%)
Stage 4	35 (5%)	19 (4.8%)
Stage 5	6 (0.8%)	0 (0%)

Evaluation of Secondary Outcomes: Chronic Kidney Disease Status, Hemoglobin Levels, Protein Levels, and Cholesterol Levels

Out of the total 690 eligible female patients included in the study, 169 did not have hemoglobin levels reported and were excluded from the analysis. Of the remaining 521, 30.7% (n=160) females had normal hemoglobin levels and 69.2% (n=361) had low hemoglobin levels (Table 3). Interestingly, when examining the association between low hemoglobin and CKD stage, we see that anemia was more prevalent in CKD stage 3a (40.4%, n=146) in CKD females than in more advanced stages (Table 3). Hemoglobin levels were not reported in 107 males, resulting in an analysis of 390 males. Anemia was present in 57.5% (n=163) of total males included, with a higher prevalence in CKD 3a (47.8%, n=78), similar to females.

**Table 3:** CKD stage and Hb levels. CKD: chronic kidney disease; NR: not reported

CKD stage	Females (N=521)		Males (N=283)			Total (N=804) (NR=276)		
	Normal=11.5-15.7 g/dL (N=160)	Low <11.5 g/dL (N=361)	Increased >15.7 g/dL (N=1)	Normal=11.5-15.7 g/dL (N=119)	Low <11.5 g/dL (N=163)	Increased >15.7 g/dL (N=1)	Normal=11.5-15.7 g/dL (N=279)	Low <11.5 g/dL (N=524)
Total	30.7%	69.2%	0.35%	42%	57.5%	0.1%	34.7%	65.1%
1	16 (10%)	18 (4.9%)	0 (0%)	12 (10.08%)	15 (9.2%)	0 (0%)	28 (10%)	33 (6.2%)
2	40 (25%)	81 (22.4%)	0 (0%)	31 (26.05%)	31 (19%)	0 (0%)	71 (25.4%)	112 (21.3%)
3a	79 (49.38%)	146 (40.4%)	1 (100%)	54 (45.38%)	78 (47.8%)	1 (0.1%)	133 (47.6%)	224 (42.7%)
3b	22 (13.75%)	90 (24.9%)	0 (0%)	16 (13.45%)	30 (18.4%)	0 (0%)	38 (13.6%)	120 (22.9%)
4	3 (1.88%)	21 (5.8%)	0 (0%)	6 (5.04%)	9 (5.5%)	0 (0%)	9 (3.2%)	30 (5.7%)
5	0 (0%)	5 (1.39%)	0 (0%)	0 (0%)	0 (0%)	0 (0%)	0 (0%)	5 (0.9%)

In conclusion, anemia was present in all stages of chronic kidney disease among females, indicating a greater severity of underlying pathologies. When considering the total population, the overall prevalence of low hemoglobin levels was 65.1% (n=524) with the highest prevalence observed in participants with CKD stage 3a (42.7%, n=224) (Table 3).

Upon analyzing protein levels, from the total number of patients included in the analysis, 133 did not have protein levels measured. From the remaining patients, it was observed that hypoproteinemia is associated with both genders (35% in females {n=208} and 34.2% in males {n=121}) and is more prevalent in CKD stage 3a (n=81 from the total females with hypoproteinemia {38.9%} and n=55 from total males with hypoproteinemia {45.4%}) (Table 4).

**Table 4:** CKD stage and protein levels. CKD: chronic kidney disease; NR: not reported

CKD stage	Females (N=594)			Males (N=353)			Total (N=947) (NR=133)		
	Increased >87 g/L	Normal=66-87 g/L	Low <66 g/L	Increased >87 g/L	Normal=66-87 g/L	Low <66 g/L	Increased >87 g/L	Normal=66-87 g/L	Low <66 g/L
Total	5 (0.8%)	381 (64.1%)	208 (35%)	1 (0.2%)	231 (65.4%)	121 (34.2%)	6 (0.6%)	612 (64.6%)	329 (34.7%)
1	0	26 (6.8%)	16 (7.6%)	0	17 (7.36%)	14 (11.5%)	0 (0%)	43 (7%)	30 (9.1%)
2	0	86 (22.5%)	46 (22.1%)	0	46 (19.9%)	28 (23.1%)	0 (0%)	132 (21.5%)	74 (22.4%)
3a	4 (80%)	166 (43.5%)	81 (38.9%)	0	114 (49.3%)	55 (45.4%)	4 (66.6%)	280 (45.7%)	136 (41.3%)
3b	0	87 (22.8%)	49 (23.5%)	1 (100%)	39 (16.8%)	22 (18.1%)	1 (16.6%)	126 (20.5%)	71 (21.5%)
4	1 (20%)	15 (3.9%)	13 (6.2%)	0	15 (6.4%)	2 (1.6%)	1 (16.6%)	30 (4.9%)	15 (4.5%)
5	0	1 (0.2%)	3 (1.4%)	0	0 (0%)	0 (0%)	0 (0%)	1 (0.1%)	3 (0.9%)

When examining total cholesterol levels, 114 patients did not have cholesterol levels measured from the total of 1080 included patients. High levels of cholesterol were observed in 26.9% (n=260) of patients included in the analysis, with the highest prevalence observed in patients with CKD 3a (44.2%, n=115). Regarding gender distribution, 31.9% (n=196) of females had high cholesterol levels, with the highest prevalence reported in stage 3a (45.4%, n=89). Regarding males, 18.1% (n=64) had high levels of cholesterol, with the highest prevalence reported in stage 3a (40%, n=26 of high cholesterol in males) (Table 5).

**Table 5:** CKD stage and cholesterol levels. CKD: chronic kidney disease; NR: not reported

CKD stage	Females (N=614)			Males (N=352)			Total (N=966) (NR=114)		
	Normal=120-200 mg/dL	Increased >200 mg/dL	Low <120 mg/dL	Normal=120-200 mg/dL	Increased >200 mg/dL	Low <120 mg/dL	Normal=120-200 mg/dL	Increased >200 mg/dL	Low <120 mg/dL
Total	360 (58.6%)	196 (31.9%)	58 (9.4%)	223 (63.3%)	64 (18.1%)	65 (18.4%)	583 (60%)	260 (26.9%)	123 (12.7%)
1	24 (6.6%)	15 (7.6%)	5 (8.6%)	20 (8.9%)	6 (9.3%)	5 (7.6%)	44 (7.5%)	21 (8%)	10 (8.1%)
2	82 (22.7%)	41 (20.9%)	14 (24.1%)	54 (24.2%)	14 (21.8%)	8 (12.3%)	136 (23.3%)	55 (21.1%)	22 (17.8%)
3a	150 (41.6%)	89 (45.4%)	27 (46.5%)	100 (44.8%)	26 (40%)	37 (56.9%)	250 (42.8%)	115 44.2%)	64 (52%)
3b	83 (23%)	43 (21.9%)	7 (12%)	39 (17.4%)	14 (21.8%)	13 (20%)	122 (20.9%)	57 (21.9%)	20 (16.2%)
4	18 (5%)	7 (3.5%)	5 (8.6%)	10 (4.4%)	4 (6.2%)	2 (3%)	28 (4.8%)	11 (4.2%)	7 (5.6%)
5	3 (0.8%)	1 (0.5%)	0 (0%)	0 (0%)	0 (0%)	0 (0%)	3 (0.5%)	1 (0.3%)	0 (0%)

## Discussion

Elderly patients frequently present with multiple comorbidities, which significantly impact both their treatment and prognosis. A patient-centered, holistic approach is therefore imperative in such cases. Notably, Romania appears to have a lower estimated prevalence of chronic kidney disease (CKD) than the European average, and there is a notable scarcity of data regarding the elderly population in Romania. However, our findings indicate a CKD prevalence of 25.03% (n=among the elderly population >65 years), when considering CKD≤60 mL/min/1.73 m^2^. When we add CKD stages 1 and 2 (elderly population with proteinuria), the prevalence of CKD increases to 34.6% (n=3116 with 1080 patients with CKD). The most prevalent stage was stage 3a (45%, n=486 of the total number of patients included in the analysis). Regarding gender distribution, in the cohort we included, there were 63.8% (n=690 females and 36.1% males {n=390}). In both genders, stage 3a was the most prevalent (43% in females {n=297} and 48.4% in males {n=189}). In reference to age distribution, 26.2% (n=283) were young elderly, 52.1% (n=563) were adult elderly, and 21.6% (n=234) were old elderly, with CKD stage 3a prevalent in all age groups. Anemia was present in 65.1% (n=524) of participants with the highest prevalence in stage 3a. Hypoproteinemia was present in 34.7% (n=329) of participants, out of which 41.3% (n=136) were in stage 3a.

In our exploration of epidemiological studies on CKD prevalence, one study conducted in Romania involving 60,000 patients yielded interesting findings regarding the association of age and gender with CKD prevalence. For individuals under 65 years, CKD was more prevalent in women, but this trend shifted after the age of 65 years, with men exhibiting a higher prevalence, which increased with advancing age. In our analysis, both males and females exhibited similar patterns of CKD stages [10].

An examination of CKD prevalence in Europe, specifically in the 65-74 years age group, reveals considerable variations. Germany, Spain, and Ireland exhibited the highest rates, ranging from 19.1% to 25.5%. In contrast, France, Italy, and Switzerland reported lower CKD prevalence, with rates of 7% and 4.1%. It's vital to note that these figures were drawn from the general population and weren't adjusted for hypertension and diabetes [4]. In our analysis, 26.2% (n=283) of patients included in our cohort were elderly (65-74 years old).

Recent research from Southern Denmark, where the average age was 76.4 years, showed CKD prevalence ranging from 4.83% to 4.98%. Interestingly, the percentage of women with eGFR <60 mL/min/1.73 m^2^ exceeded that of the general population. However, the incidence of end-stage CKD was higher among men, possibly due to delayed medical attention-seeking. The study also highlighted a link between low socioeconomic status and CKD prevalence [12].

Regarding gender analysis, two separate studies conducted in the United States (2008) and Japan (2020) demonstrated that males have a higher risk of developing advanced stages of CKD, while females exhibit a higher prevalence of other CKD stages [13,14]. A 2019 meta-analysis, comprising 30 studies, supported the notion that CKD progression is linked with male gender. However, it is crucial to consider that non-biological factors, such as lifestyle, culture, and socioeconomic conditions may influence this association. In our analysis, females with CKD were more prevalent (63.8% females and 36.1% males).

In contrast to the global average, Romania exhibits the highest CKD prevalence among patients aged 65 years and above, surpassing even Germany, which was previously considered to have the highest prevalence. Our analysis shows a 25.03% (n=780) prevalence of CKD stages 3a-5 and a total prevalence of CKD of 34.6% (n=1080). Several factors may account for this finding, including a lower socioeconomic status compared to other countries, the absence of a CKD screening program, and a low referral rate for screening among patients, even though the nephrology clinic that serves this region is located in the same hospital as the geriatric and gerontology department, with which we maintain close collaboration. Additionally, during the pandemic, the geriatric and gerontology department became a support clinic for infected patients, and data from this period are insufficient.

In a study published in 2015, in participants with stage 3 CKD, arterial hypertension was the most prevalent comorbidity (87.8%), while ischemic heart disease was present in 22.9% of participants and heart failure in only 3.5% of cases, unlike our cohort, where heart failure was most prevalent [15]. In a Hungarian cohort that included almost 10000 participants with CKD stages 3a-5, arterial hypertension was also the most prevalent comorbidity, present in 70.2% of cases, followed by diabetes (41.5%) and heart failure (20.5%) [16].

It is known that aging is accompanied by a physiologic decline of renal function. Our data shows an overall prevalence of CKD of 34.6% in an elderly population (age >65 years). The trend is lower in our population compared to other global data that show a prevalence of CKD of 44% in the elderly population [17]. A nationwide cross-sectional study in Croatia found an overall CKD prevalence of 17.1% across all stages, with a median age of 72 years among individuals with CKD [18]. A retrospective study conducted in Poland analyzed 352 biopsies from patients aged ≥65 years. The most prevalent diagnoses were membranous nephropathy, focal segmental glomerulosclerosis, and amyloidosis, compared to biopsies in a younger population. Additionally, proteinuria was the predominant manifestation in the elderly population [18]. In our study, the prevalence of CKD stages 1 and 2 with proteinuria was 9.6%. Additionally, the prevalence of CKD is higher in Eastern Europe compared to the United States and Western European countries and this likely relates to the higher prevalence of risk factors and the lower economic status in Eastern Europe [19].

This study has several strengths. Firstly, it is an inaugural study exclusively focusing on CKD among the elderly. Secondly, it is one of the most extensive studies conducted in Romania. Additionally, it was carried out in a university center serving a population of 4.5 million patients, featuring a dedicated nephrology clinic. This report delves into the prevalence of CKD within the elderly demographic, examining its correlation with essential biomarkers of malnutrition (protein levels and cholesterol levels). The study spans a decade, from 2012 to 2022, focusing on Romania's northeastern region, specifically Moldova. This is the second substantial analysis carried out in Romania and Eastern Europe, with a unique emphasis on patients aged 65 years and older. Another strength of our study is that the participants included did not have a prior diagnosis of CKD. The research is conducted in the northeastern region of Romania, a socioeconomically deprived area. This regional focus provides valuable data on CKD prevalence in an underserved population, contributing to a broader understanding of the disease in different socioeconomic contexts. The study adds valuable data to the limited pool of research on CKD prevalence in Romania and Eastern Europe. This regional contribution is important for understanding global health patterns and for informing local healthcare policies.

However, the study has certain limitations that must be acknowledged. A reduced number of patients was included in the analysis for the period from 2020 to 2022 due to the shift towards COVID-19 support measures. Additionally, nearly half of the initial determinations were excluded from the study due to multiple hospitalizations during the study period or missing data for analysis. Economic status and CKD prevalence were not evaluated in comparison to other common comorbidities such as hypertension or diabetes, which are known triggers for CKD. Furthermore, it is important to note that all determinations relied on creatinine levels, which can underestimate eGFR in the elderly, who are more susceptible to sarcopenia. We used the CKD-EPI 2021 equation based on creatinine levels as the reference for evaluating renal function, as cystatin-C-based equations are not currently reimbursed by the National Insurance policy. The study also lacked assessment measures for cognitive status, malnutrition, depressive disorder, or functional status, which represents a significant limitation. Another limitation is that CKD trends were impacted by the COVID-19 pandemic, as the hospital ward was repurposed to support COVID-19 patients, leading to insufficient data during this period. Additionally, we recognize the potential overestimation of representativeness in this study. The geriatric department functions as both a tertiary hospital and a university clinic in the area; however, not all patients may be referred to the department. This limits the study’s ability to provide a continuous analysis over the entire decade.

## Conclusions

Our research revealed a total CKD prevalence of 25.03% in stages 3a-5 in the study population and 9.6% for CKD stages 1 and 2. When considering age distribution, it became evident that CKD had a higher incidence in elderly participants. CKD was more prevalent in females in the cohort we included in our analysis. In both genders, stage 3a was the most prevalent (43% in females and 48.4% in males). Improving CKD in elderly patients requires a comprehensive approach focusing on slowing progression, managing symptoms, and enhancing quality of life. Screening for CKD in the elderly is essential for early detection and management, as kidney function naturally declines with age, increasing the risk of complications. Additionally, the elderly population has multiple comorbidities that contribute to the additional risk of developing CKD.

This is the first study in Romania to examine CKD prevalence in the elderly population. Our findings indicate a high overall CKD prevalence (34.6%) across all stages, aligning with data from other Eastern European countries. The most prevalent stage in both genders was stage 3a. Nevertheless, it's important to emphasize the need for further evaluation to establish more robust conclusions. More extensive studies and in-depth analysis are required to confirm these trends and provide a solid foundation for making informed clinical decisions. A more detailed exploration of contributing factors, comorbidities, and potential interventions is essential to form more conclusive and actionable insights. Moreover, there is an inconsistency regarding the diagnosis of CKD among epidemiological studies.
